# Effectiveness of a Smartphone App–Based Intervention With Bluetooth-Connected Monitoring Devices and a Feedback System in Heart Failure (SMART-HF Trial): Randomized Controlled Trial

**DOI:** 10.2196/52075

**Published:** 2024-04-29

**Authors:** Minjae Yoon, Seonhwa Lee, Jah Yeon Choi, Mi-Hyang Jung, Jong-Chan Youn, Chi Young Shim, Jin-Oh Choi, Eung Ju Kim, Hyungseop Kim, Byung-Su Yoo, Yeon Joo Son, Dong-Ju Choi

**Affiliations:** 1 Division of Cardiology, Department of Internal Medicine Seoul National University Bundang Hospital, Seoul National University College of Medicine Seognam Republic of Korea; 2 Division of Cardiology, Department of Internal Medicine Keimyung University Dongsan Hospital Daegu Republic of Korea; 3 Cardiovascular Center Korea University Guro Hospital, Korea University College of Medicine Seoul Republic of Korea; 4 Division of Cardiology, Department of Internal Medicine Seoul St. Mary's Hospital, Catholic Research Institute for Intractable Cardiovascular Disease, College of Medicine The Catholic University of Korea Seoul Republic of Korea; 5 Division of Cardiology, Department of Internal Medicine Severance Hospital, Yonsei University College of Medicine Seoul Republic of Korea; 6 Cardiac and Vascular Center Samsung Medical Center, Sungkyunkwan University Seoul Republic of Korea; 7 Department of Internal Medicine Wonju Severance Christian Hospital, Yonsei University Wonju College of Medicine Woonju Republic of Korea; 8 Healthcare Business Department, AI/DX Convergence Business Group KT Seoul Republic of Korea

**Keywords:** heart failure, mobile applications, mobile health, self-care, vital sign monitoring, mobile phone

## Abstract

**Background:**

Current heart failure (HF) guidelines recommend a multidisciplinary approach, discharge education, and self-management for HF. However, the recommendations are challenging to implement in real-world clinical settings.

**Objective:**

We developed a mobile health (mHealth) platform for HF self-care to evaluate whether a smartphone app–based intervention with Bluetooth-connected monitoring devices and a feedback system can help improve HF symptoms.

**Methods:**

In this prospective, randomized, multicenter study, we enrolled patients 20 years of age and older, hospitalized for acute HF, and who could use a smartphone from 7 tertiary hospitals in South Korea. In the intervention group (n=39), the apps were automatically paired with Bluetooth-connected monitoring devices. The patients could enter information on vital signs, HF symptoms, diet, medications, and exercise regimen into the app daily and receive feedback or alerts on their input. In the control group (n=38), patients could only enter their blood pressure, heart rate, and weight using conventional, non-Bluetooth devices and could not receive any feedback or alerts from the app. The primary end point was the change in dyspnea symptom scores from baseline to 4 weeks, assessed using a questionnaire.

**Results:**

At 4 weeks, the change in dyspnea symptom score from baseline was significantly greater in the intervention group than in the control group (mean –1.3, SD 2.1 vs mean –0.3, SD 2.3; *P*=.048). A significant reduction was found in body water composition from baseline to the final measurement in the intervention group (baseline level mean 7.4, SD 2.5 vs final level mean 6.6, SD 2.5; *P*=.003). App adherence, which was assessed based on log-in or the percentage of days when symptoms were first observed, was higher in the intervention group than in the control group. Composite end points, including death, rehospitalization, and urgent HF visits, were not significantly different between the 2 groups.

**Conclusions:**

The mobile-based health platform with Bluetooth-connected monitoring devices and a feedback system demonstrated improvement in dyspnea symptoms in patients with HF. This study provides evidence and rationale for implementing mobile app–based self-care strategies and feedback for patients with HF.

**Trial Registration:**

ClinicalTrials.gov NCT05668000; https://clinicaltrials.gov/study/NCT05668000

## Introduction

Heart failure (HF) is a rapidly growing public health problem, with an estimated prevalence of 64 million people globally [1-5]. Although outcomes of HF have recently improved with the development of medications, a high rate of readmission remains after initial hospitalization for acute HF [6-10]. Recent studies have shown that 18%-23% of patients with acute HF were readmitted within 1 month, and these numbers have not decreased recently [6,11,12]. Therefore, the postdischarge management of patients with HF is important. In particular, multidisciplinary interventions, in addition to pharmacotherapy and discharge education, are known to improve quality of life and reduce hospitalizations [13-16]. A recent meta-analysis has shown that self-management interventions, including symptom and sign monitoring, education, and enhancement of drug adherence, improve outcomes of HF-related hospitalization and all-cause death, despite heterogeneity in the interventions [17]. Thus, current HF guidelines recommend multidisciplinary management, specific discharge education, and support to facilitate HF self-care [18-20].

Although encouraging self-care and providing feedback from health care providers are the most effective means, they are difficult to implement in a real-world clinical setting. These are also associated with higher costs and require more infrastructure and manpower. Smartphones are currently available to most of the general population at an affordable cost. The advent of mobile health (mHealth) technology and advances in artificial intelligence (AI) have enabled patient self-care, symptom, and sign monitoring, as well as mobile-based feedback via smartphone apps. Recent studies using smartphone apps for the self-management of cardiovascular disease demonstrated that this intervention led to better outcomes than the control groups [21-23]. Additionally, some recent studies and meta-analyses regarding mHealth apps have supported self-care among patients with HF [24-30]. However, these previous studies had a small number of participants and limited app functionality. Self-monitoring and feedback are particularly important, and the lack of these critical features limits the interpretation of the effectiveness of HF apps. Furthermore, functions provided in previous mobile-based interventions for HF were heterogeneous and varied between studies, which may lead to inconsistent results.

Given the uncertainty regarding the benefits of mobile apps for HF and their diverse functionality, we developed a mHealth platform to provide self-management interventions for patients with HF. This study aimed to evaluate whether this smartphone app–based intervention with Bluetooth-connected monitoring devices and a feedback system could improve the symptoms and clinical outcomes of HF.

## Methods

### Study Design and Population

The self-monitoring using a mobile app to improve symptoms and reduce rehospitalization and mortality in heart failure (SMART-HF) study is a prospective, multicenter, randomized, open-label trial to evaluate the efficacy of smartphone apps in improving the symptoms and outcomes of HF. A total of 7 tertiary university hospitals in South Korea participated in this study. The study design was registered at ClinicalTrials.gov (NCT05668000).

We enrolled patients aged older than 20 years who were hospitalized for acute HF with obvious symptoms or signs of HF at admission. Patients with N-terminal pro-B-type natriuretic peptide (NT-proBNP) levels ≥400 pg/mL or brain natriuretic peptide levels ≥100 pg/mL were enrolled. Since smartphone use was essential in this study, participants should be able to use Android smartphones well and be capable of following instructions on how to use apps. Patients with a baseline systolic blood pressure (BP) of <90 mm Hg or a resting heart rate (HR) of <50 beats per minute were excluded from this study. Additionally, patients were excluded if they had a cardiac [30] implantable electronic device that could interfere with body water analysis. Patients who were expected to have a prolonged hospital stay owing to medical problems other than HF were also excluded. The detailed inclusion and exclusion criteria are presented in Table S1 in Multimedia Appendix 1.

### Ethical Considerations

This clinical trial was approved by the institutional review boards of Seoul National University Bundang Hospital (B-2211-795-304) and other hospitals. The study was conducted in accordance with the Declaration of Helsinki, and all study data were deidentified. All patients provided written informed consent upon enrollment.

### Patient Recruitment and Randomization

**Figure 1:**
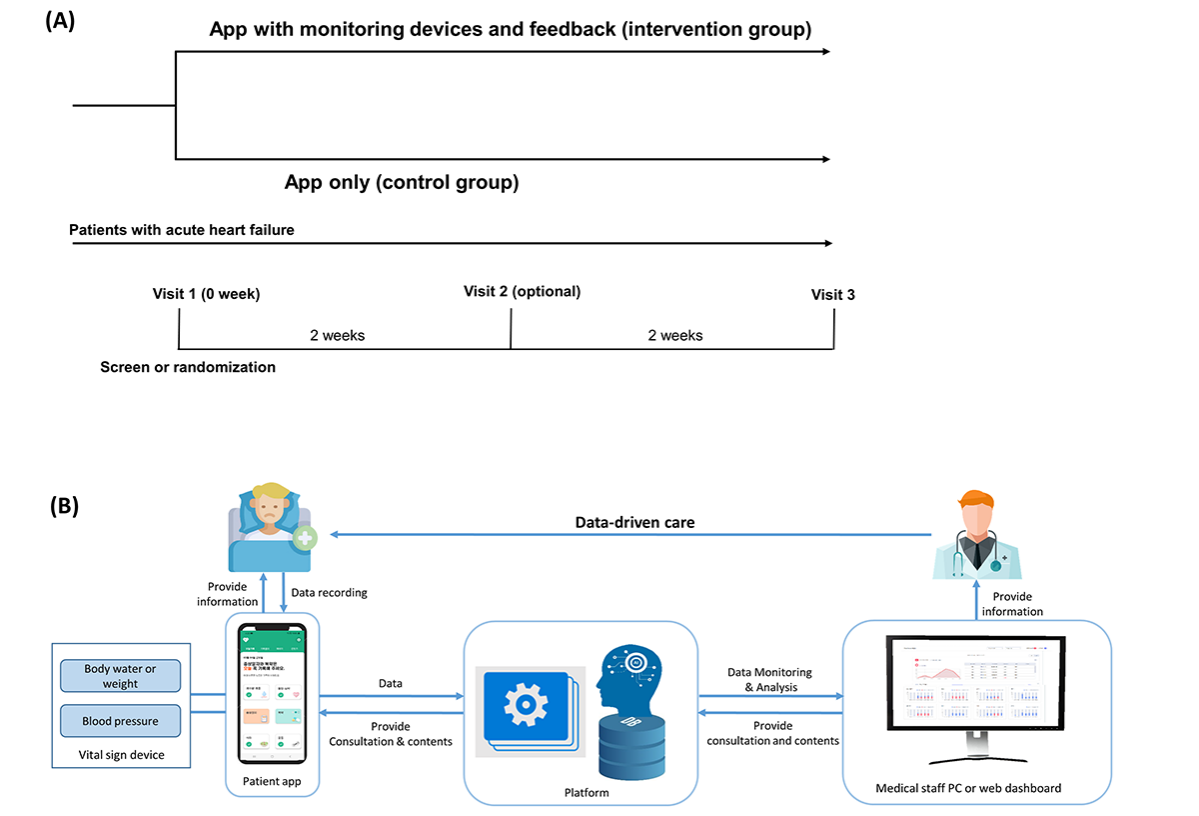
Study design of the (A) SMART-HF trial and (B) system of app and platform. (A) Patients with acute HF were randomized into the intervention (app with monitoring devices and feedback) or control (app only) groups. (B) The app and platform are comprised of three parts (1) a smartphone app for patients, (2) a dashboard system for physicians, and (3) a clinical decision support system on the platform. PC: personal computer; SMART-HF: self-monitoring using a mobile app to improve symptoms and reduce rehospitalization and mortality in heart failure.

After a comprehensive interview, eligible participants were asked to provide written informed consent before being discharged from the hospital. At baseline (visit 1), all participants were assessed for sex, age, demographics, vital signs, comorbidities, laboratory data, and medication use. Eligible participants were randomized 1:1 to either the intervention group (app with Bluetooth-connected monitoring devices and feedback) or the control group (app only) using a web-based central randomization service (Figure 1A). Due to the nature of the intervention, the study participants and investigators interacting with the patients were not blinded to the group allocation.

### Intervention and Follow-Up

We developed a mobile app and operating platform to improve the symptoms and outcomes of patients with HF. The app and platform comprised three parts: (1) a smartphone app for patients, (2) a dashboard system for physicians, and (3) a clinical decision support system (CDSS) on the platform (Figure 1B and Figures S1 and S2 in Multimedia Appendix 1).

**Figure 2:**
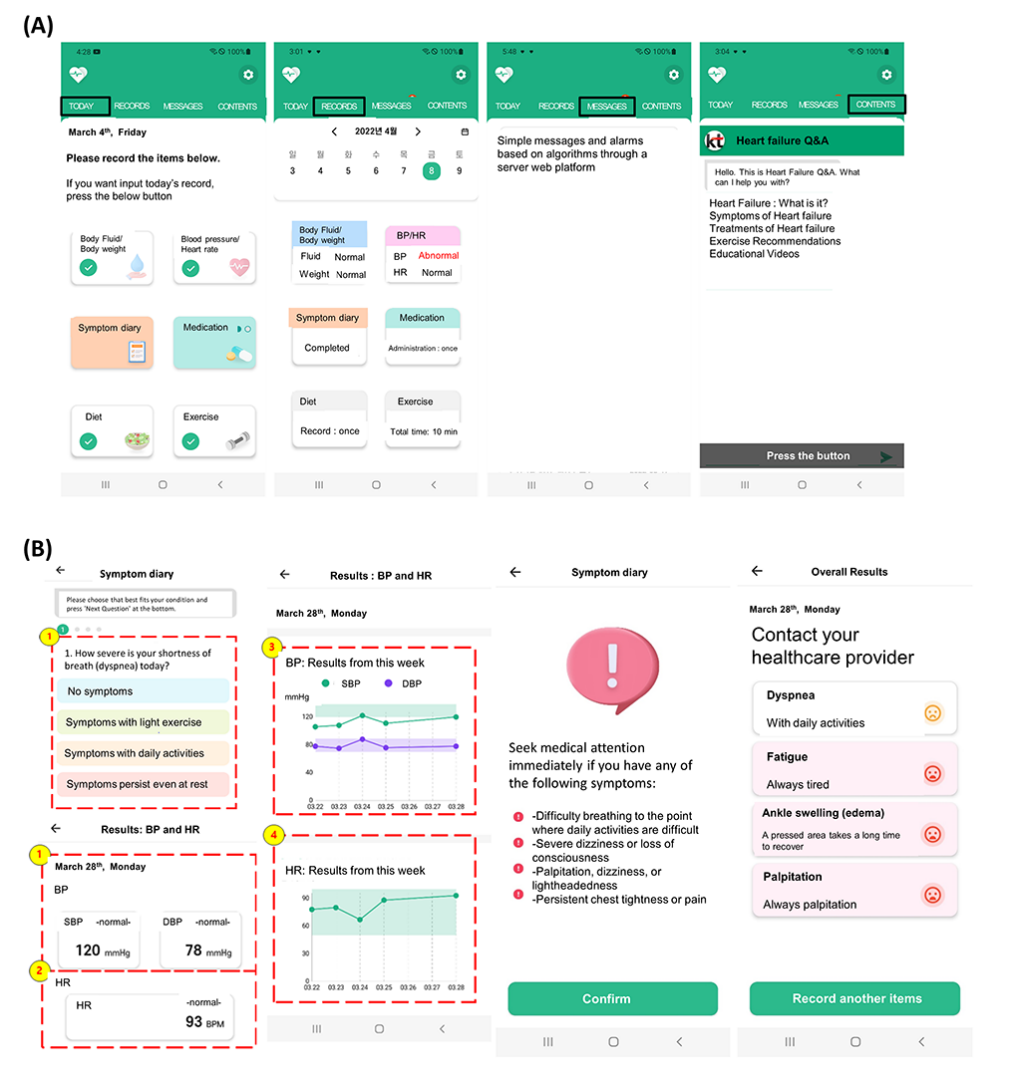
Application menu screens and functions. (A) Menu screens and (B) application functions. BP: blood pressure; DBP: diastolic blood pressure; HR: heart rate; SBP: systolic blood pressure.

The smartphone app comprised 4 menu screens (Figure 2), and the detailed functions of the app are described in Table S2 in Multimedia Appendix 1. In the “Today” menu, patients can enter their information, including body water, weight, BP, HR, symptom diary, medications, diet, and exercise. Patients can input their symptoms of HF, including dyspnea, fatigue, ankle edema, and palpitations, daily. Symptoms were scored as 0 (no symptoms), 1 (mild symptoms), 2 (moderate symptoms), and 3 (severe symptoms; Table S3 in Multimedia Appendix 1). Moreover, they could enter their daily diet or physical activity into the apps, and the amount of sodium in their diet was assessed through a camera image using AI (Figure S3 in Multimedia Appendix 1). Additionally, the BP, HR, weight, and body water could be entered into the app by the patients (intervention + control group) or automatically paired using Bluetooth-connected monitoring devices (intervention group only). In the “Records” menu, patients can access and review their historical data entered by date, as well as receive feedback regarding medication adherence, dietary habits, and exercise. Furthermore, the app can analyze and evaluate a patient’s symptom scores or measured vital signs and subsequently generate alerts based on a predefined algorithm (Tables S3 and S4 in Multimedia Appendix 1) using the CDSS. In the event of rapid changes in a patient’s symptoms or vital signs, the app prompts the patient to provide information regarding medication adherence, symptom severity, and any other concerns. Upon perceiving significant changes, it sends a message to the patients, advising them to contact their health care provider. In the “Contents” menu, the app can provide patients with information about HF, including symptoms, treatment, dietary guidelines, and exercise recommendations using chatbots.

The dashboard system was designed to present stored information to physicians (Figure S4 in Multimedia Appendix 1). This information included BP, HR, body weight, body water composition, HF symptom score, medications, diet, exercise, and any data entered by the patient. Physicians could easily observe the vital sign trends (daily, weekly, and monthly) of the participants. The CDSS analyzes the input data and generates recommendations for users using AI and algorithms. For example, it aggregates data and information from the patient and sends necessary messaging alerts to the patient’s app and, if needed, to the health care provider’s dashboard during office visits.

After randomization, both groups installed the study app on their Android smartphones, which the research nurse provided. Only the intervention group had full access to all functions of the app, and the apps were automatically paired with Bluetooth-connected monitoring devices, including a BP monitor, weight scale, and body water analyzer. The intervention group received feedback and alerts from the app. In the control group, patients could enter their BP, HR, and weight into the app using conventional non-Bluetooth devices only. Additionally, they received no feedback or alerts from the app. The “Contents” menu providing information about HF was available in both groups. The study aimed to evaluate the improvement in HF symptoms based on the functionality of the mobile app between the intervention and control groups, with a particular focus on Bluetooth-connected monitoring devices and the feedback function of the app.

We used a Bluetooth-enabled BP monitor BP170 (InBody Co). Bioimpedance analysis was performed using a portable multifrequency bioimpedance device (BWA ON; InBody Co) to analyze body water composition. Bioimpedance analysis has recently proven its efficacy in patients with acute HF with dyspnea [31], and the device can estimate body fluid status using extracellular water divided by total body water of the 4 limbs and trunk. The parameters of extracellular water divided by total body water <0.390, 0.390-0.400, and >0.400 were considered to be “normal,” “slightly over,” and “over,” respectively (Table S5 in Multimedia Appendix 1). The device classifies body water levels from levels 1 to 16.

Follow-up visits were scheduled at 2 (visit 2, optional) and 4 (visit 3) weeks after randomization. Both groups received standard care according to the current HF guidelines [20,32], and HF medication could be modified at the discretion of the treating physicians.

### Study Outcomes

The primary end point of the study was the change in dyspnea symptom scores from baseline to 4 weeks, which was assessed using a questionnaire. We used a visual analog scale and a numerical rating scale for dyspnea ranging from 0 to 10 based on previous studies [33,34] (Figure S5 in Multimedia Appendix 1).

There were two secondary end points: (1) a composite outcome, including death, rehospitalization, and urgent visit for HF and (2) the change in body water composition from baseline to the last measurement in the intervention group. The exploratory clinical outcome was adherence to the app, which was calculated using either log-in access logs or the percentage of days when symptoms were entered into the app. App adherence was defined as follows:

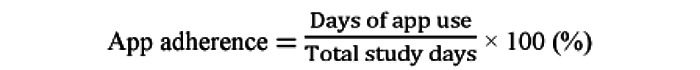



We also compared app satisfaction scores at 4 weeks through a questionnaire, ranging from 0 to 10, between the intervention and control groups. If patients could not attend the scheduled study date, the outcome measures were assessed telephonically.

### Sample Size and Statistical Analysis

Due to the absence of previous research and the pioneering nature of our intervention, our study adopts an exploratory approach. The primary objective was not to test a specific hypothesis regarding efficacy but rather to collect preliminary data and assess feasibility in preparation for a larger-scale study. Consequently, a sample size calculation was not conducted at this preliminary stage. Since this was a pilot study to explore the benefits of mobile apps for HF, we enrolled a total of 84 participants across 7 institutes, considering the duration of the study and the number of participating hospitals.

Categorical variables are reported as frequencies (percentages), and continuous variables are expressed as means (SD) or medians with IQR. Categorical variables were compared using the Pearson chi-square test or Fisher exact test, and continuous variables were compared using the Student *t* test or the Mann-Whitney *U* test. The change in body water composition from baseline to the last measurement after randomization was analyzed using the paired *t* test.

The intention-to-treat analysis included all randomized patients. The efficacy end points were primarily analyzed using the full analysis set, which included randomized participants who used the mobile app at least once. We also performed a per-protocol sensitivity analysis, including patients who completed the study protocol. For patients who dropped out before the end of the trial or had missing data, we used the latest available records for analysis, namely, the last observation carried forward method.

All tests were 2-tailed, and a *P* value <.05 was considered statistically significant. Statistical analyses were performed by using R (version 4.2.0; R Core Team).

## Results

### Patient Enrollment and Clinical Characteristics

**Figure 3:**
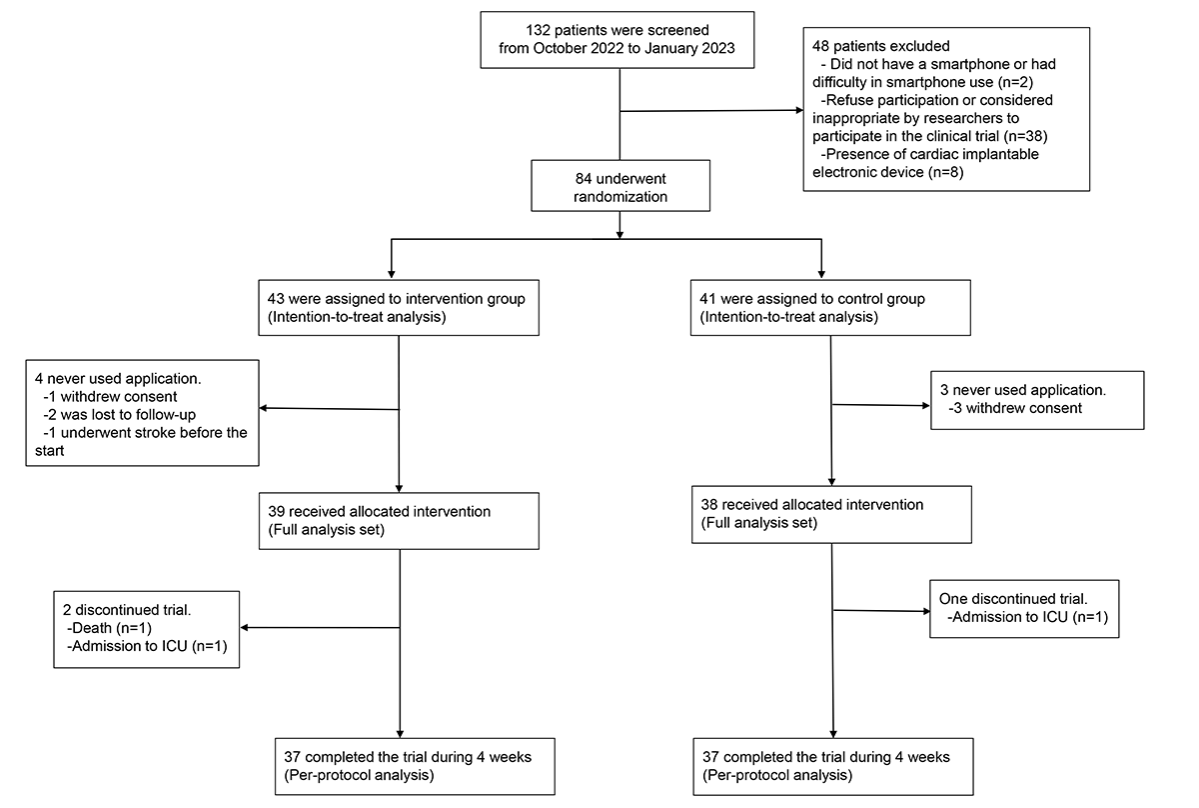
CONSORT flow diagram. CONSORT: Consolidated Standards of Reporting Trials; ICU: intensive care unit.

From October 2022 to January 2023, 132 patients from 7 centers were screened for eligibility, and 84 were randomly assigned to the intervention (n=43) or control (n=41) group (Figure 3 and CONSORT [Consolidated Standards of Reporting Trials] checklist in Multimedia Appendix 2). After allocation, 7 patients (4 and 3 in the intervention and control groups, respectively) were excluded because they did not use the app at least once; thus, 77 patients (39 and 38 in the intervention and control groups, respectively) were included in the full analysis set.

The baseline characteristics of the groups are presented in Table 1. The mean age of the total population was 62.1 (SD 14.7) years; 62% were male, 61% had de novo HF, and 30% had HF of an ischemic etiology. The median left ventricular ejection fraction was 38.7% (IQR 28%-58%) and the median NT-proBN*P* value was 2572 (IQR 1211-6651) pg/mL. The baseline characteristics, including age, sex, demographic data, laboratory values, and comorbidities, were well balanced between the 2 groups, except for diastolic BP. Additionally, the medication use at discharge was not significantly different between the 2 groups.

**Table 1:** Baseline characteristics of the patients according to the treatment groups (full analysis set).

Variables			Intervention (n=39)	Control (n=38)	*P* value
**Clinical characteristics**					
	Age (years), mean (SD)		58.4 (16.0)	65.7 (12.6)	.06
	Age ≥65 years, n (%)		16 (41	19 (50)	.57
	Male, n (%)		25 (64)	23 (61)	.93
	Height (cm), mean (SD)		164.5 (8.8)	163.3 (9.6)	.59
	Weight (kg), median (IQR)		63.0 (57.0-75.1)	61.0 (52.6-71.4)	.16
	BMI (kg/m²), median (IQR)		24.1 (21.4-27.3)	23.9 (20.6-25.5)	.25
	Systolic blood pressure (mm Hg), mean (SD)		124.1 (16.7)	117.8 (17.6)	.11
	Diastolic blood pressure (mm Hg), mean (SD)		76.4 (10.9)	69.1 (11.7)	.006
	De novo HF^a^, n (%)		23 (59)	24 (63)	.89
	Ischemic etiology, n (%)		12 (31)	11 (29)	>.99
	Hypertension, n (%)		18 (46)	12 (32)	.28
	Diabetes mellitus, n (%)		14 (36)	15 (40)	.93
	Chronic kidney disease, n (%)		9 (23)	5 (13)	.44
	Atrial fibrillation, n (%)		13 (33)	16 (42)	.58
	**NYHA^b^ functional class (n=36), n (%)**				.78
		I	3 (8)	4 (11)	
		II	20 (56)	23 (64)	
		III	11 (31)	8 (22)	
		IV	2 (6)	1 (3)	
	LVEF^c^, median (IQR)		39.7 (30.1-58.5)	34.7 (25.0-56.0)	.27
**Laboratory test**					
	Hemoglobin (g/dL), mean (SD)		12.5 (2.5)	12.7 (2.6)	.63
	eGFR^d^ (mL/minute/1.73 m^2^), median (IQR)		78.0 (51.5-94.0)	74.0 (53.8-91.0)	.84
	NT-proBNP^e^ (pg/mL; n=74), median (IQR)		2866 (1333-8198)	2058 (911-6651)	.30
**Medication use at discharge, n (%)**					
	RAS^f^ inhibitor		26 (67)	27 (71)	.87
	Beta-blocker		29 (74)	26 (68)	.75
	Mineralocorticoid receptor antagonist		20 (51)	26 (68)	.19
	SGLT2^g^ inhibitor		17 (44)	14 (36)	.71
	Loop diuretic		23 (59)	31 (82)	.06

^a^HF: heart failure.

^b^NYHA: New York Heart Association.

^c^LVEF: left ventricular ejection fraction.

^d^eGFR: estimated glomerular filtration rate.

^e^NT-proBNP: N-terminal pro-B-type natriuretic peptide.

^f^RAS: renin-angiotensin system.

^g^SGLT2: sodium-glucose cotransporter 2.

### Dyspnea Symptom Score

**Figure 4:**
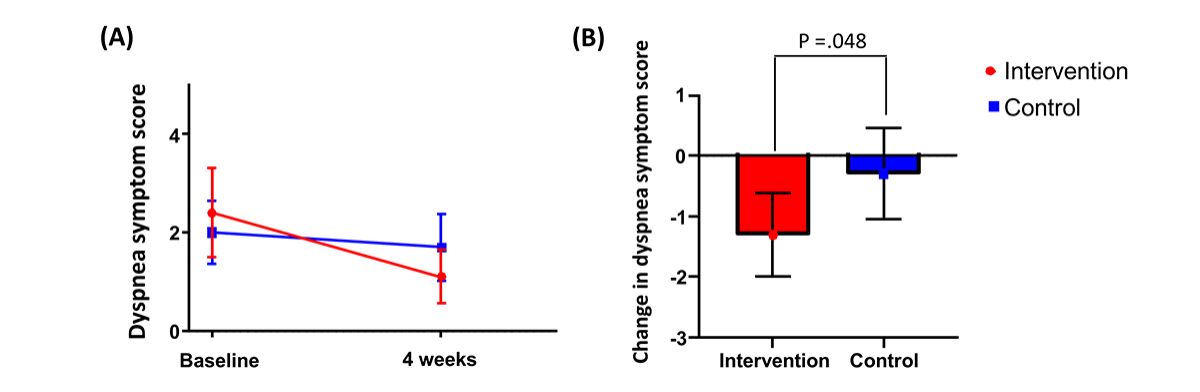
Change in dyspnea symptom score in the intervention and control groups. (A) Dyspnea score during the trial follow-up. (B) Change in dyspnea score from baseline to 4 weeks. The error bars represent the 95% CI.

The dyspnea symptom scores assessed using the questionnaire are presented in Table 2 and Figure 4. The baseline dyspnea symptom scores were not significantly different between the 2 groups (intervention vs control: mean 2.4, SD 2.8 vs mean 2.0, SD 2.0; *P*=.46). Regarding the primary end point, change in dyspnea symptom score from baseline to 4 weeks was significantly greater in the intervention group than in the control group (mean –1.3, SD 2.1 vs mean –0.3, SD 2.3; *P*=.048).

**Table 2:** Dyspnea symptom scores, obtained using the questionnaire, according to the treatment groups (full analysis set).

Variables	Intervention (n=39), mean (SD)	Control (n=38), mean (SD)	Absolute difference (95% CI)	*P* value
Dyspnea symptom scores at baseline	2.4 (2.8)	2.0 (2.0)	0.5 (–0.7 to 1.5)	.46
Dyspnea symptom scores at 4 weeks	1.1 (1.7)	1.7 (2.1)	–0.6 (–1.5 to 0.3)	.19
Change in dyspnea symptom scores from baseline to 4 weeks	–1.3 (2.1)	–0.3 (2.3)	–1.0 (–2.0 to 0.0)	.048

### Secondary End Points

**Figure 5:**
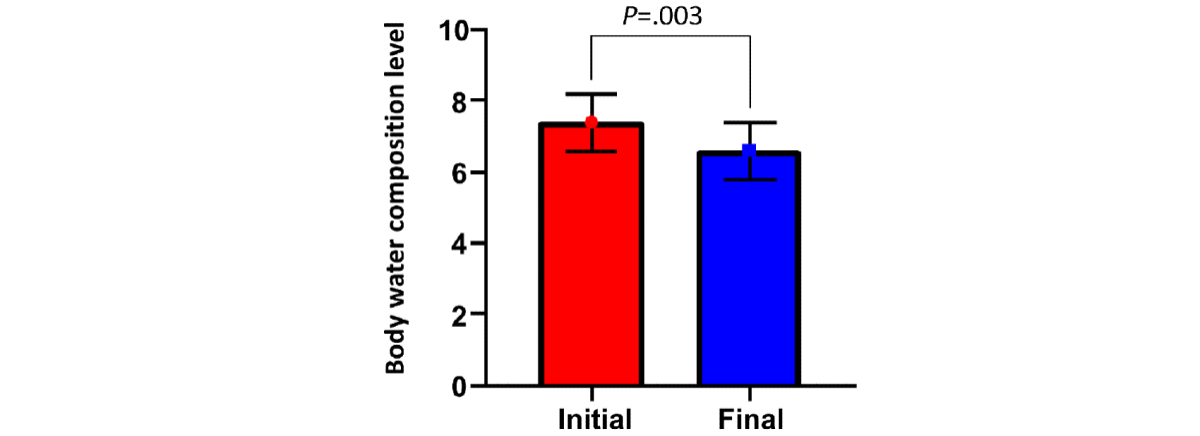
Change in body water composition level from baseline to last measurement in the intervention group.

Secondary composite end points, including death, rehospitalization, and urgent visit for HF, were not significantly different between the 2 groups (3/39 vs 3/38; *P*>.99). A significant reduction was found in the level of body water composition from baseline to the last measurement in the intervention group (baseline level mean 7.4, SD 2.5 vs final level mean 6.6, SD 2.5; *P*=.003; Figure 5).

### App Adherence and Patient Satisfaction

**Figure 6:**
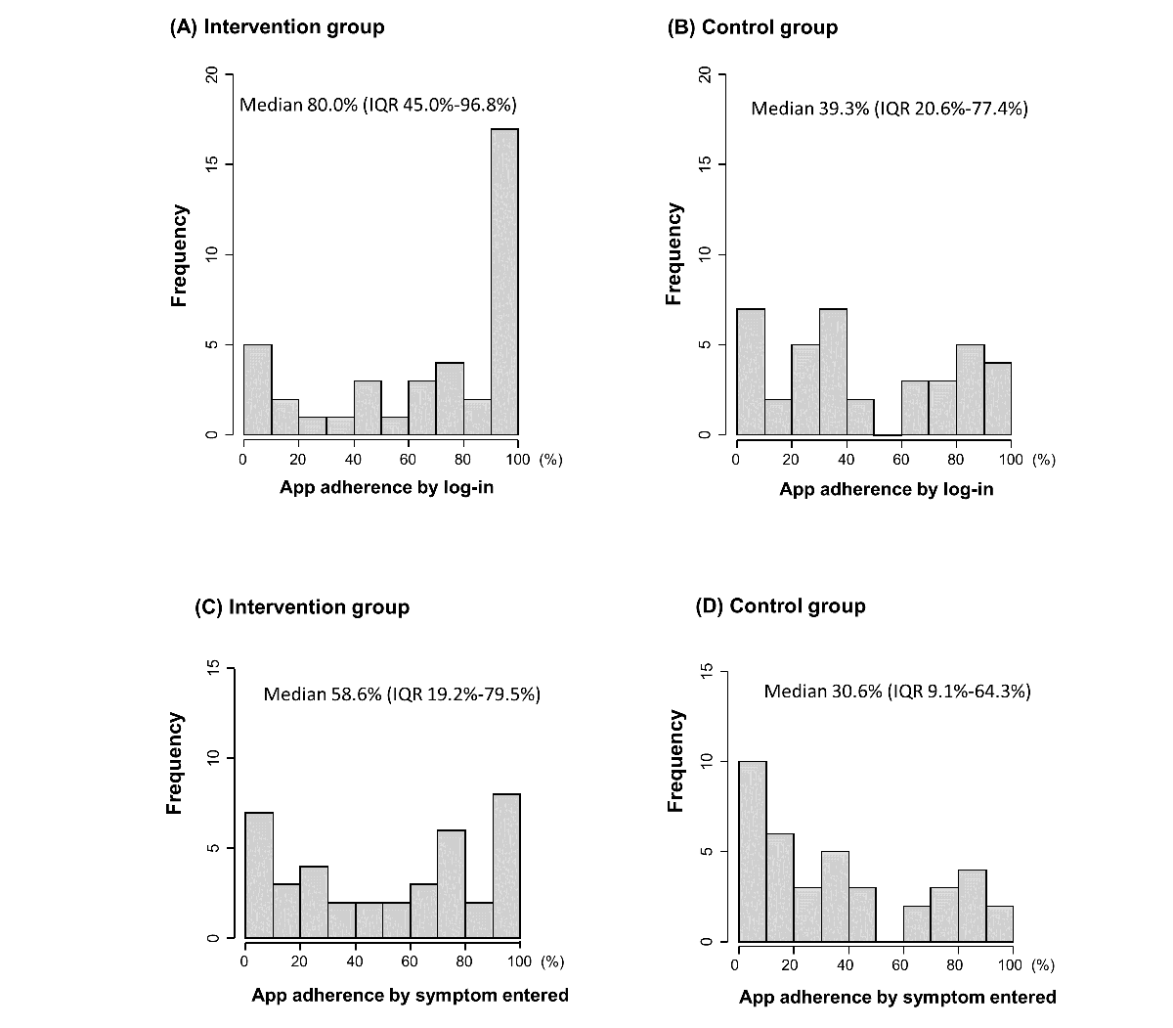
Distribution of app adherence according to the treatment groups. App adherence was assessed based on log in in (A) the intervention and (B) control groups. App adherence evaluated based on the percentage of days when symptoms were entered into the app in (C) the intervention and (D) control groups. IQR, interquartile range.

The distribution of app adherence among the treatment and control groups is presented in Figure 6. App adherence, which was assessed based on log-in access logs was higher in the intervention group than in the control group (80.0%, IQR 45.0%-96.8% vs 39.3%, IQR 20.6%-77.4%; *P*=.003). Additionally, the app adherence evaluated based on the percentage of days when symptom was entered into the app was significantly higher in the intervention group than in the control group (58.6%, IQR 19.2%-79.5% vs 30.6%, IQR 9.1%-64.3%; *P*=.046).

We also analyzed differences in app adherence according to age. Patients aged 65 years and older showed lower app adherence, as indicated by log-in access logs than those aged younger than 65 years in the overall population (45.9%, IQR 8.9%-79.2% vs 74.9%, IQR 35.7%-95.5%; *P*=.02). The intervention group showed similar results (61.4%, IQR 13.5%-89.5% vs 92.9%, IQR 64.2%-98.5%; *P*=.04); however, in the control group, no significant difference was found in app adherence according to the age cutoff of 65 years (39.4%, IQR 5.7%-76.7% vs 39.3%, IQR 27.8%-74.6%; *P*=.40).

App satisfaction scores at 4 weeks tended to be higher in the intervention group than in the control group; however, the difference was not statistically significant (mean 7.1, SD 2.6 vs mean 6.2, SD 2.9; *P*=.15).

### Sensitivity Analysis

Overall, 74 patients (37 in the intervention group and 37 in the control group) completed the trial over 4 weeks (per-protocol analysis; Figure 3). Table S6 and Figure S6 in Multimedia Appendix 1 show the results of sensitivity analyses of the per-protocol population. There was a trend toward a greater reduction in dyspnea symptom scores from baseline to 4 weeks in the intervention group than in the control group, similar to the primary analyses (mean –1.4, SD 2.1 vs mean –0.5, SD 2.1; *P*=.08). Regarding body water composition, the intervention group showed a greater reduction in the level of body water composition from baseline to last measurement, consistent with the main analysis (baseline level mean 7.4, SD 2.5 vs final level mean 6.6, SD 2.6; *P*=.006).

## Discussion

### Principal Results

We developed a smartphone app and mHealth platform for patients with HF. The main findings of this study are (1) a mobile-based health platform with Bluetooth-connected monitoring devices and feedback improved dyspnea symptoms compared with the control group and (2) this app-based intervention led to an improvement in the level of body water composition and higher adherence to the app. We believe that the findings of this prospective, randomized study will help in formulating public health strategies to improve HF outcomes using smartphones.

Support for facilitating self-care is crucial for patients with HF [18-20]. However, proper patient self-care and delivery of health care provider feedback are challenging to implement in a real-world clinical setting because of the required time, financial, and human resources. It is challenging for patients to know how to respond to changes in their symptoms or vital signs. It is also impossible for medical staff to quickly check patient information or provide feedback 24 hours a day, 7 days a week. These disadvantages can be overcome by self-management interventions via smartphones, including self-care, symptom and sign monitoring, and mobile-based feedback, which are easily accessible anytime, anywhere. Considering these advantages of a smartphone app–based intervention, including cost-effective and accessible methods, our group has previously developed mobile app platforms for patients with atrial fibrillation [35,36] or hypertension [37]. Therefore, we planned to develop an app for patients with HF.

Our mobile app for HF provides diverse functions using the concept of mHealth platforms for personalized interventions. Vital signs and body water can be paired with Bluetooth-connected devices, and patients can enter their HF symptoms into the apps. Patients can also receive feedback or alerts from the app based on the information they have entered. Additionally, patients can obtain information about HF using chatbots. Although direct interaction between patients and health care providers may be an ideal situation, it may not be feasible owing to rising costs and a shortage of medical staff. Our app-based monitoring and feedback system had the advantage of being accessible and quick for patients to receive feedback promptly on their changes without high personnel or cost. Although patients in the control group had access to the smartphone app for HF self-care, the improvement in dyspnea symptoms was more pronounced in the intervention group than in the control group. Patients in the control group could only use the limited functionality of the app, were unable to use Bluetooth devices, and could not receive any feedback or alerts. Therefore, our study suggests that the comprehensive functionality of apps, especially in the monitoring and feedback system, is particularly important for their role.

### Comparison With Prior Work

Some previous studies have focused on mHealth apps for HF [24-30]. However, these studies were limited by the small number of participants and limited app functionality. Additionally, previous mobile apps have heterogeneous functions and platforms [24,25], and this diversity might have influenced the benefits of mobile apps in HF. Considering the inconsistent benefits of mobile apps in HF [25], we developed a mobile app with multiple functions in collaboration with 1 of the largest high-tech information technology companies in Korea (KT). In our opinion, this app has a considerably wider range of features and is more efficiently organized than other existing apps [28-30]. Our app has the advantage of keeping patients engaged in their condition and self-management after leaving the hospital. Not only does this provide psychological reassurance, but it also serves the patient’s best interests by keeping a close eye on any changes in their clinical presentation or symptoms. Patients can assess their symptoms more frequently, make lifestyle changes, or contact health care providers earlier. The dashboard system also enables health care professionals to identify changes in a patient’s condition quickly. Furthermore, the app contains elements that may be of interest to patients, including sodium analysis in food using AI and various HF information. Overall, we assumed that the monitoring and feedback function and app adherence are especially important for the mobile app’s role in HF and believe that the benefits of our app could improve the symptoms of HF.

Increasing app adherence has been a common challenge in many studies. For example, a recent study showed that only approximately 30% of patients actively used the HF app, underscoring the importance of caution regarding the enrollment of critically ill, postacute, and older patients [30]. In our study, median adherence to apps based on log-in access logs was 80% and 39% in the intervention and control groups, respectively, which we believe should be further improved. In the intervention group, we believe that a more comprehensive functionality of apps, including Bluetooth-connected monitoring devices and a feedback system, may lead to higher adherence to apps than in the control group. Additionally, this disparity in app functionality may be associated with a higher trend in app satisfaction scores within the intervention group.

Particularly, as a large proportion of patients with HF tend to be older and app adherence appears to decline in those aged 65 years and older in our study, increasing adherence to apps for HF is crucial; this can be achieved by improving the platform to make it more user-friendly for older patients. Interestingly, at the cutoff age of 65 years, a difference was observed in app adherence according to age category in the intervention group but not in the control group, probably due to overall higher app adherence in the intervention group. In the subgroup analysis, no difference was found in the change of dyspnea symptom scores between the intervention and control groups according to an app adherence cutoff of 50%. This is probably due to the small sample size. Therefore, further research with larger study populations is warranted to confirm whether the frequency of app usage directly affects the outcomes.

### Limitations and Strengths

This study had some limitations. First, our results are limited by the small sample size of the study. Second, we only enrolled patients who had smartphones and could use them; therefore, the results of our trial may not be applicable to patients who cannot use smartphones, such as very old adults. Since a large proportion of patients with HF are older and have difficulty using smartphones, excluding them from participation may lead to bias and study limitations. Third, the true engagement and use frequency of the apps and the real responses of participants to feedback could not be analyzed. There is a lack of clarity on exactly how the frequency of app use affects clinical outcomes. Additionally, there was a relatively large number of older patients, and adherence to the app declined toward the end of the study. These limitations are commonly observed in studies that use mobile apps. Fourth, we evaluated the dyspnea symptom score using a visual analog scale and a numerical rating scale ranging from 0 to 10. However, at baseline, the average of these scores was <3 points, suggesting that this study was conducted on patients who already had achieved significant improvement in their dyspnea, which limits surveying on a scale of 0-10 over 4 weeks. Fifth, the HF medications could be modified at the discretion of each physician, which could be a confounding factor. Sixth, baseline diastolic BP was higher in the intervention group than in the control group, while patients in the intervention group tended to be younger. This difference in baseline characteristics may limit the generalizability of our conclusions. Seventh, the follow-up period in our studies was only 4 weeks, which was shorter than that in a similar study [29]; therefore, the effect of mobile apps on patients’ symptoms for a long-term follow-up period was unknown. Furthermore, secondary clinical outcomes, including death, rehospitalization, and urgent visits for HF, were not significantly different between the 2 groups in this study, although a similar study using apps [28] for HF showed reduced hospitalization for HF by mobile app. Finally, we tested a single app, and our results may not be generalizable to other smartphone-based apps for patients with HF.

Despite these limitations, the major strength of this study is the use of a mobile app with various functions developed in collaboration with a cutting-edge technology company. We believe that the features of our app can be effective solutions for improving self-care in patients with HF.

### Conclusions

The smartphone app–based intervention with Bluetooth-connected monitoring devices and feedback improved dyspnea symptoms among patients with HF compared with the control group. Considering the high cost of classical patient-provider interventions, self-care, and feedback through mobile apps are promising alternatives. Therefore, our study provides evidence and rationale for mobile app–based self-care and feedback for patients with HF.

## Appendix

Multimedia Appendix 1 App characteristics and functionalities, and participants’ clinical characteristics and outcomes.

Multimedia Appendix 2 CONSORT-EHEALTH checklist.

## Abbreviations

AI: artificial intelligence
BP: blood pressure
CDSS: clinical decision support system
CONSORT: Consolidated Standards of Reporting Trials
HF: heart failure
HR: heart rate
mHealth: mobile health
NT-proBNP: N-terminal pro-B-type natriuretic peptide
SMART-HF: self-monitoring using a mobile app to improve symptoms and reduce rehospitalization and mortality in heart failure

## Data Availability

The data sets generated and analyzed during this study are available from the corresponding author upon reasonable request.
