# Supplementary material for: Effectiveness of a Smartphone App–Based Intervention With Bluetooth-Connected Monitoring Devices and a Feedback System in Heart Failure (SMART-HF Trial): Randomized Controlled Trial
Source: J Med Internet Res. 2024 Apr 29;26:e52075. doi: 10.2196/52075 (PMC11091801; doi:10.2196/52075)
Supplement: Multimedia Appendix 1 [file jmir_v26i1e52075_app1.docx]

**Table S1. Inclusion and exclusion criteria**

| ***Inclusion criteria:*** |
| --- |
| 1. ≥20 years of age, male or female  2. Patients who were hospitalized for acute HF with obvious symptoms or signs of HF at admission  3. Patients with NT-proBNP levels ≥400 pg/mL or BNP levels ≥100 pg/mL  4. Patients who can use Android smartphone well and follow the instructions on how to use the app in Korean language |
| ***Exclusion criteria:*** |
| 1. Patients with systolic blood pressure < 90 mmHg, or resting heart rate < 50 beats/min at screening visit  2. Patients with cardiac implantable electronic device which interfere with body water analysis  3. Patients with a history of the following diseases  -hypertrophic cardiomyopathy with left ventricular outflow tract obstruction, cor pulmonale, history of cardiac arrest, and severe cerebrovascular events including ischemic stroke and hemorrhagic stroke within 6 months  4. Patients who are expected to have prolonged hospital stay owing to medical problems other than HF (for example, femoral neck fracture)  5. Patients with a history of alcohol or drug abuse  6. Patients who cannot use smartphone  7. Patients who are judged as both legally and psychologically inadequate to participate in the clinical study by the investigator |

BNP, B-type natriuretic peptide; HF, heart failure; NT-proBNP, N-terminal pro B-type natriuretic peptide

**Table S2. Application menu screens and funciton**

| App menu | Description |  |
| --- | --- | --- |
| Today | Record of eight items: body water, weight, blood pressure, heart rate, symptom diary, medications, diet, and exercise. |  |
| Records | Review ‘Today's Record’ and check the results for history by date. Review recommendation alerts. |  |
| Messages | Integrating and analyzing the measured information and data to deliver simple messages and warnings based on algorithms through a server web platform |  |
| Contents | Chatbot functionality for providing answers to patient queries and delivering knowledge  Content for patients with heart failure: symptoms, treatment, dietary guidelines, exercise recommendations, etc. |  |
| Function | Description | |
| Body water/Weight measurement | -Measurement using a Bluetooth device, with automatic storage of records  -Tracking the important physiological changes in heart failure and providing appropriate encouragement and alerts | |
| Blood pressure/Heart rate measurement | -Measurement using a Bluetooth blood pressure/heart rate monitoring device, with automatic storage of records  -Tracking the important physiological changes in heart failure patients and providing appropriate encouragement and warnings | |
| Symptom checklist | -Checking and recording symptoms in four categories  -Tracking changes in symptoms in patients with heart failure and providing appropriate encouragement and warnings | |
| Medication | -Check the intake of heart failure medications and provide a reminder alarm if not taken | |
| Diet | -Recording consumed food by KT artificial intelligence food tag, and indicating excessive sodium intake in the food consumed  -Providing appropriate encouragement and warnings | |
| Exercise | -Measuring activity details and duration to provide appropriate exercise levels | |

**Table S3. Scores of heart failure symptoms and change in vital signs**

| Heart failure symptoms | | | | | |
| --- | --- | --- | --- | --- | --- |
|  | Items/scores | 0 | 1 | 2 | 3 |
| 1 | Dyspnea | No symptoms | Symptoms with light exercise | Symptoms with daily activities | Symptoms present even at rest |
| 2 | Fatigue | No symptoms | Occasionally tired | Frequently tired | Always tired |
| 3 | Ankle swelling (edema) | No symptoms | Slight swelling when pressed | Significant swelling when pressed | Pressed area takes a long time to recover |
| 4 | Palpitation | No symptoms | Occasional palpitation | Frequent palpitation | Always palpitation |
| Change in vital signs^a^ | | | | | |
|  | Items/scores | 0 | 1 | 2 | 3 |
| 1 | Reduced SBP | Reduced less than 5 mmHg | Reduced more than 5 mmHg | Reduced more than 10 mmHg | Reduced more than 15 mmHg |
| 2. | Increased SBP | Reduced less than 10 mmHg | Reduced more than 10 mmHg | Reduced more than 15 mmHg | Reduced more than 20 mmHg |
| 3 | Decreased heart rate | Reduced less than 5 bpm | Reduced more than 5 bpm | Reduced more than 10 bpm | Reduced more than 20 bpm |
| 4 | Increased heart rate | Increased less than 5 bpm | Increased more than 5 bpm | Increased more than 10 bpm | Increased more than 20 bpm |
| 5. | Weight change | Reduced, or increased less than 0.5kg | Increased more than 0.5 kg | Increased more than 1.0 kg | Increased more than 1.5 kg |

The total score is calculated as the sum of the individual scores.

^a^Comparison with previous measurements for each item.

SBP, systolic blood pressure.

**Table S4. Message alarm algorithm**

|  | Conditions | Message |
| --- | --- | --- |
| 1 | Total score of 6 or more, but less than 9 | Are you experiencing more severe shortness of breath and swelling than before? Please check if you are taking medication properly, overexerting yourself or stressed, or taking any medicines other than the prescribed treatment. |
| 2 | Total score of 9 or more | Suspected worsening of the condition. Please consult with a healthcare professional or seek medical attention promptly. |
| 3 | If the heart rate is less than 50 bpm or exceeds 140 bpm | If you experience symptoms such as dizziness or shortness of breath, contact your healthcare provider. |

After making a judgment, the message content in the application is updated at the top of the screen.

**Table S5. Body water criteria using ECW/TBW ratio**

| **ECW/TBW range** | **Criteria** | **Level** |
| --- | --- | --- |
| 0.400 ≤ECW/TBW | Over | 10–16 |
| 0.390 ≤ECW/TBW ≤0.400 | Slightly over | 8–9 |
| ECW/TBW <0.390 | Normal | 1–7 |

ECW: extracellular water, TBW: total body water.

**Table S6. Dyspnea symptom scores by questionnaire according to treatment groups (per-protocol analysis)**

|  | **Intervention**  **(n = 37)** | **Control**  **(n = 37)** | **Absolute difference (95% CI)** | ***P* value** |
| --- | --- | --- | --- | --- |
| Dyspnea symptom scores at baseline | 2.4 ± 2.9 | 2.1 ± 2.0 | 0.3 (-0.8 to 1.5) | 0.57 |
| Dyspnea symptom scores at 4 weeks | 1.0 ± 1.6 | 1.6 ± 2.0 | -0.6 (-1.4 to 0.3) | 0.18 |
| Change in dyspnea symptom scores from baseline to 4 weeks | -1.4 ± 2.1 | -0.5 ± 2.1 | -0.9 (-1.9 to 0.1) | 0.08 |

Values are expressed as mean ± standard deviation or difference (95% CI).

CI, confidence interval;

**Figure S1. Hardware system diagram**


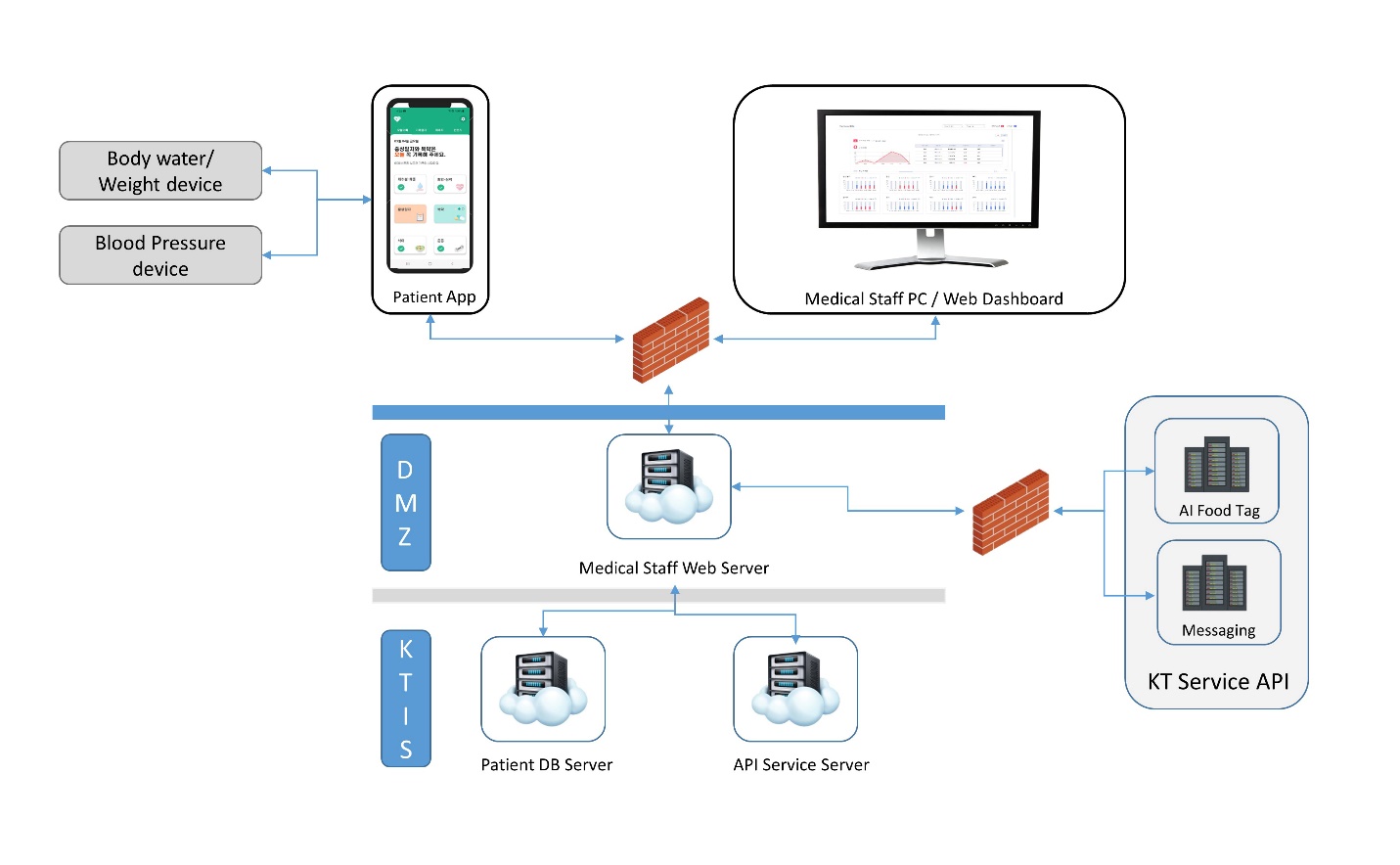


API, application programing interface, DB: database, PC, personal computer.

**Figure S2. Software system diagram**

**
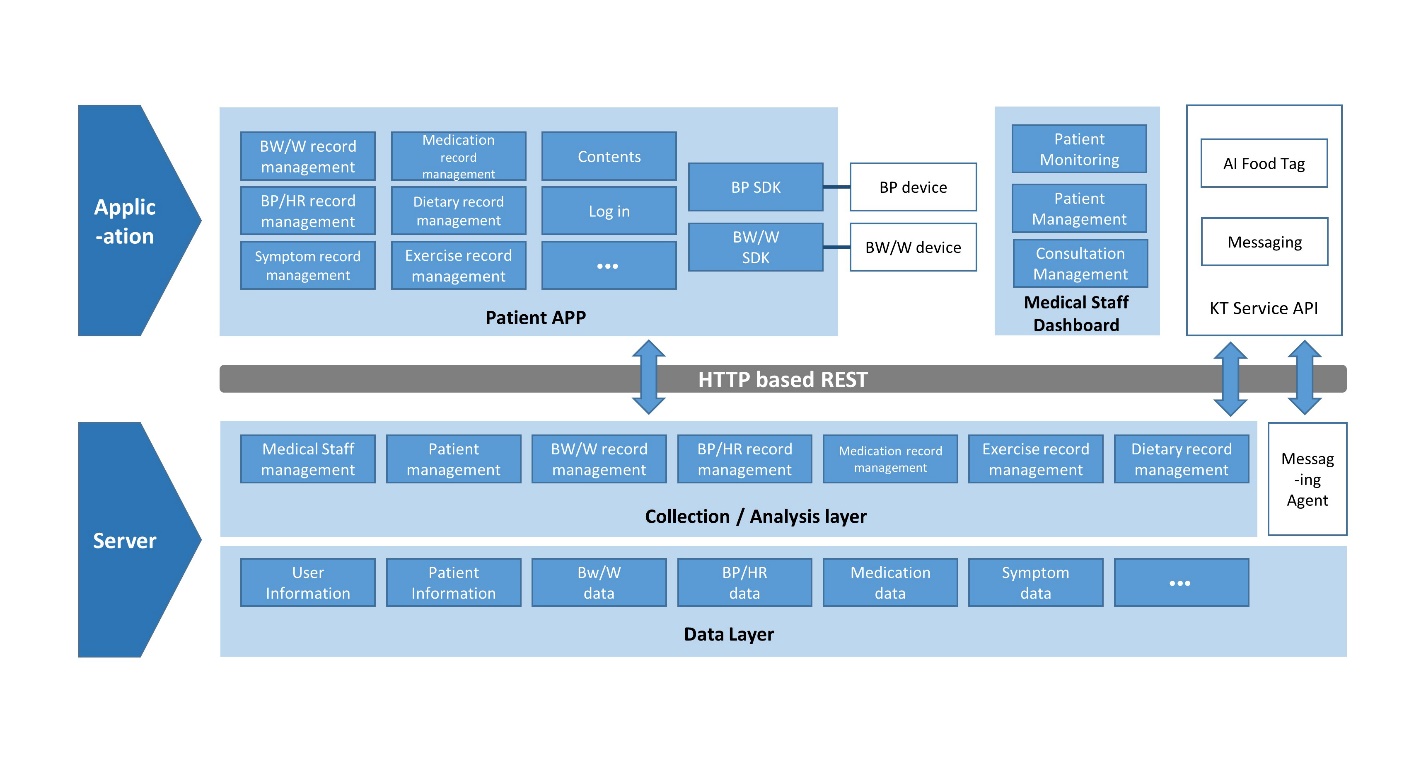
**

AI, artificial intelligence; API, application programing interface, BP: blood pressure, BW: body water, HR: heart rate, SDK: software development kit, REST: representational state transfer, W: weight.

**Figure S3. Technology that uses AI to recognize food from photos captured by smartphone**


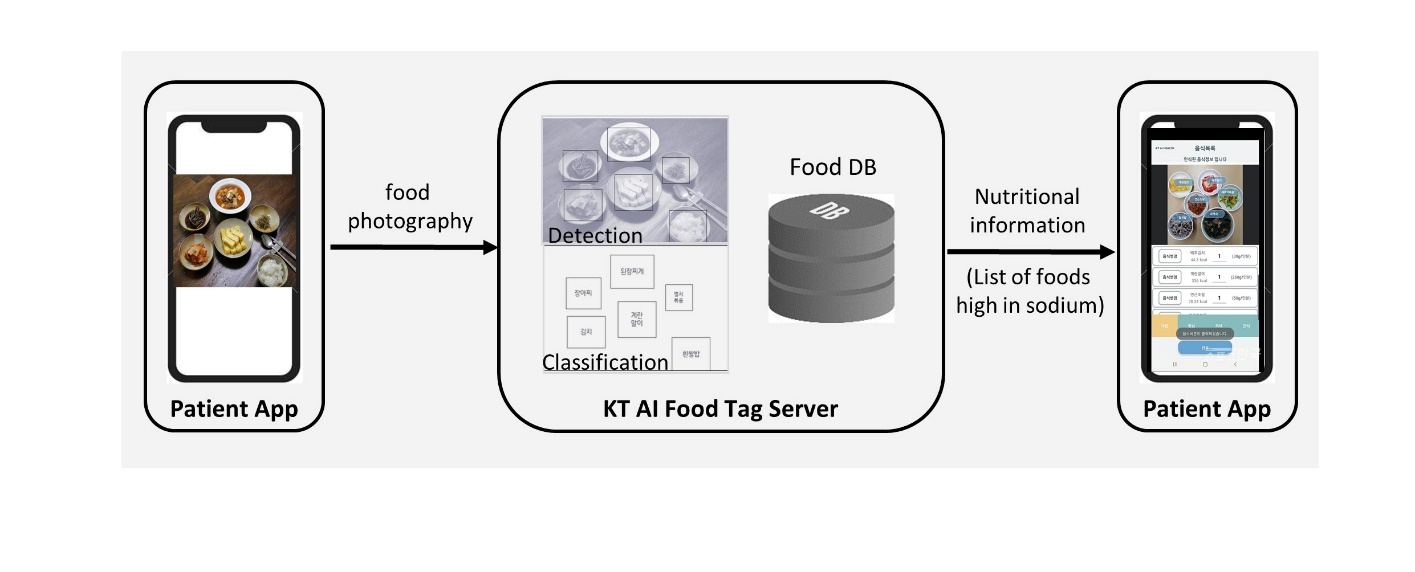


‘KT AI Food Tag’, an AI-based dietary management solution, has been certified for AI quality and testing by the Korean Artificial Intelligence Certification Center (KORAIA CC).

AI: artificial intelligence, DB, database.

**Figure S4. Dashboard system for physicians**


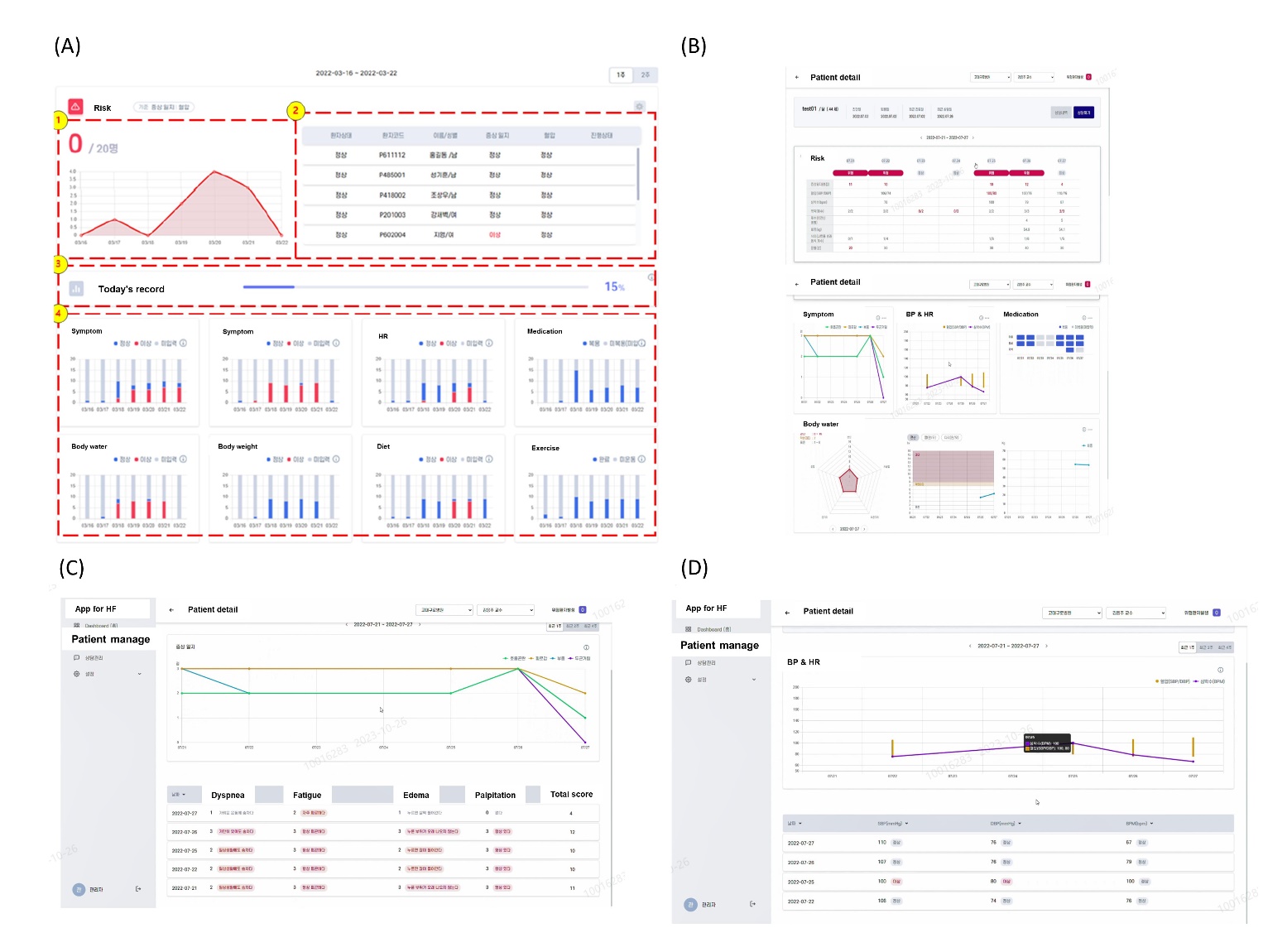


(**a**) Main screen, (**b**) individual patient information, (**c**) heart failure symptom score trend, and (**d**) vital sign trend.

**Figure S5. Visual analogue scale and numerical rating scale for dyspnea obtained using the questionnaire**


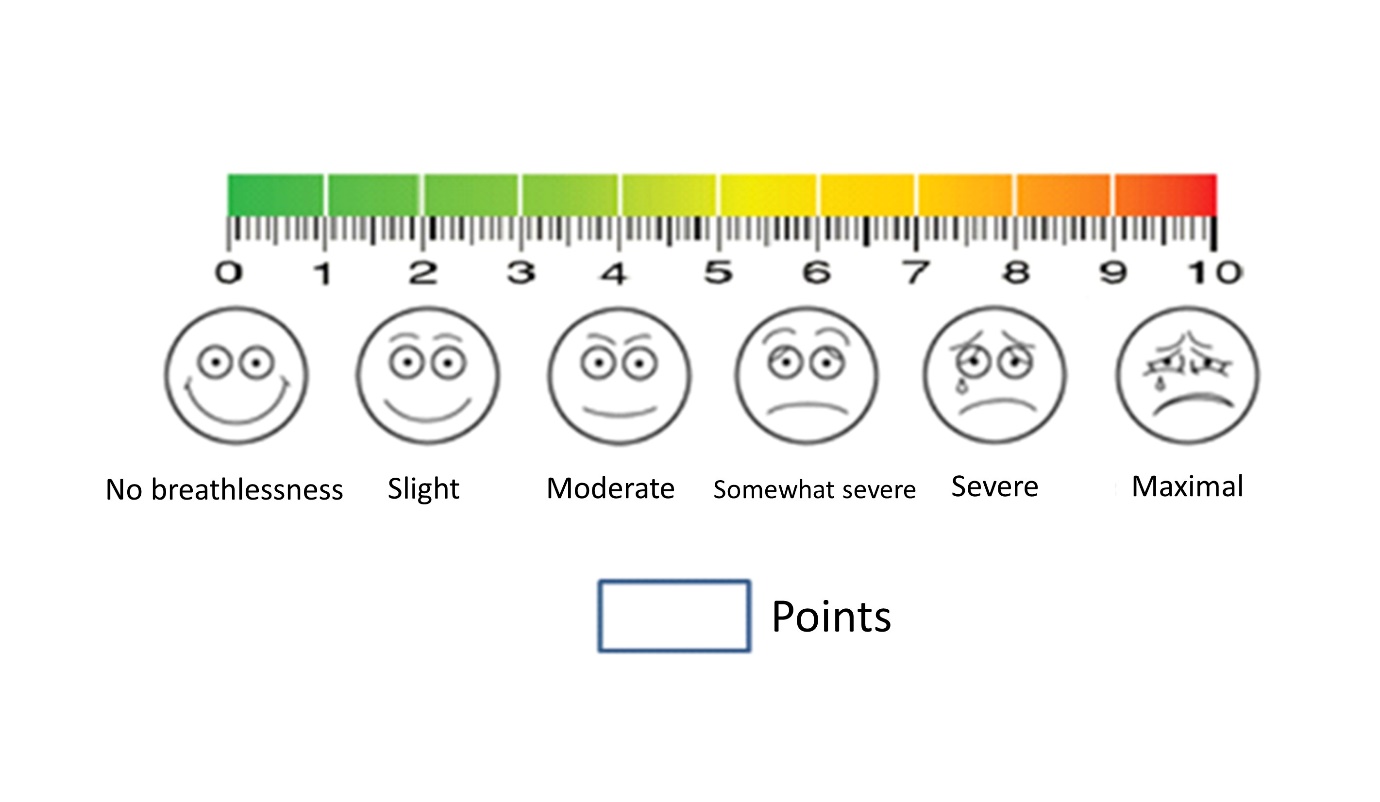


We used a visual analogue scale and a numerical rating scale for dyspnea ranging from 0 to 10.

**Figure S6. Change in dyspnea symptom score in the intervention and control groups (per-protocol analysis)**


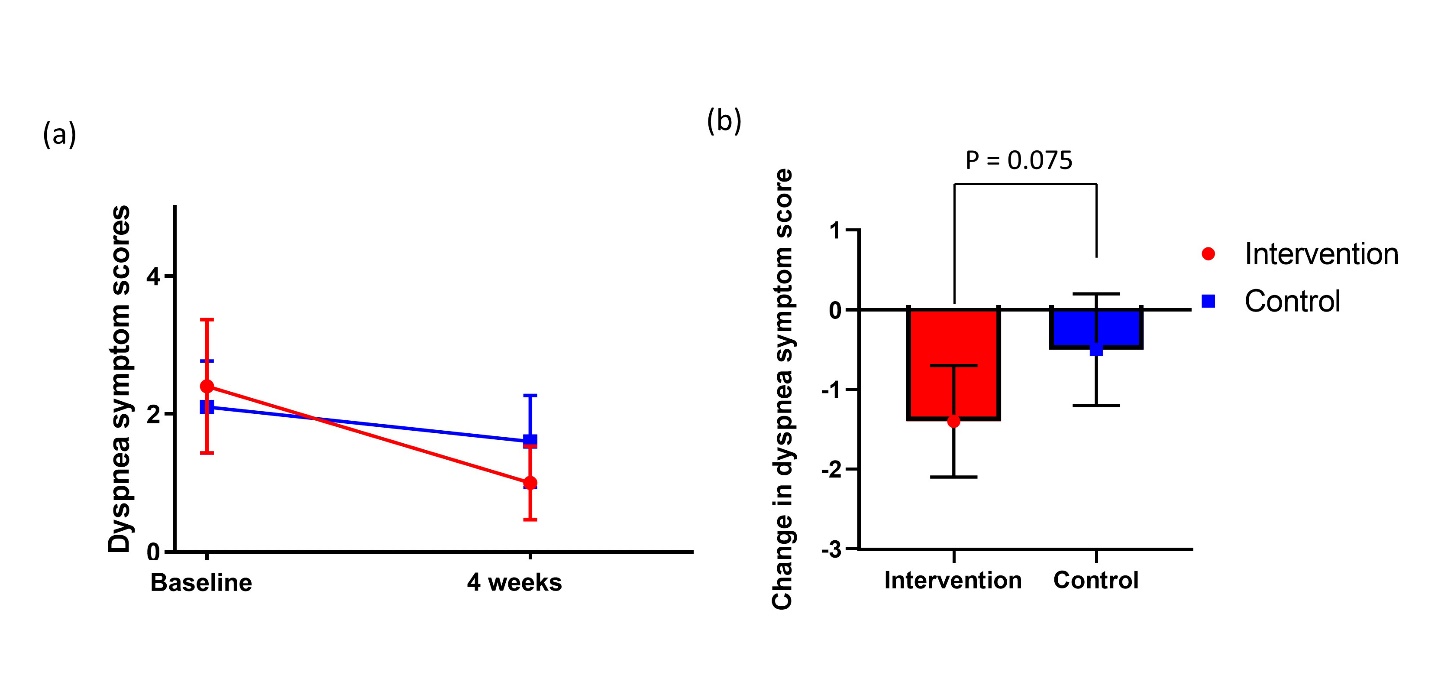


(**a**) Dyspnea score during the trial follow-up (**b**) Change in dyspnea score from baseline to 4 weeks.

The error bars represent the 95% confidence interval.
